# Reactive Oxygen Species Induce Endothelial Differentiation of Liver Cancer Stem-Like Sphere Cells through the Activation of Akt/IKK Signaling Pathway

**DOI:** 10.1155/2020/1621687

**Published:** 2020-10-10

**Authors:** Zhengbin Zhao, Jing Gao, Caili Li, Xiaoli Xu, Yihuan Hu, Shuangsheng Huang

**Affiliations:** ^1^Department of Infectious Diseases, The First Hospital of Lanzhou University, Lanzhou 730000, China; ^2^Hospital of Northwest Minzu University, Lanzhou 730030, China; ^3^Medical College of Northwest Minzu University, Lanzhou 730030, China; ^4^School of Basic Medicine, Lanzhou University, Lanzhou 730000, China

## Abstract

Cancer stem cells (CSCs) from various cancers are able to transdifferentiate into endothelial cells and further form functional blood vessels, indicating another possible resistance mechanism to antiangiogenic agents. However, it remains unclear whether CSCs from hepatocellular carcinoma have the ability to differentiate into endothelial cells, and thus resulting in resistance to antiangiogenic therapy targeting VEGF. Reactive oxygen species (ROS) are involved in the self-renewal and differentiation of CSCs, yet, their role in endothelial differentiation of CSCs has been poorly understood. In this study, we found that cancer stem-like sphere cells enriched from human hepatocellular carcinoma cell line Hep G2 could differentiate into endothelial cells morphologically and functionally, and this process could be blocked by Akt1/2 kinase inhibitor and IKK-*β* inhibitor BAY 11-7082 but not by Bevacizumab, a VEGFA-binding antibody, and DAPT, a *γ*-secretase inhibitor. Both hydrogen peroxide and BSO (an inhibitor of GSH biosynthesis) induce the differentiation of cancer stem-like sphere cells into endothelial cells, which can be canceled by the antioxidant N-Acetyl-L-cysteine (NAC). We also found that hydrogen peroxide or BSO induces the phosphorylation of Akt and IKK of endothelial differentiated sphere cells. Accordingly, both Akt1/2 kinase inhibitor and BAY 11-7082 inhibited hydrogen peroxide and BSO-mediated endothelial differentiation of cancer stem-like sphere cells. Collectively, the results of the present study demonstrate that cancer stem-like sphere cells from Hep G2 are able to differentiate into endothelial cells both morphologically and functionally, and this process is independent of VEGF and NOTCH signaling but dependent on the activation of Akt and IKK. ROS promote endothelial differentiation of cancer stem-like sphere cells through activation of Akt/IKK signaling pathway. Therefore, our study reveals a novel mechanism of resistance to conventional antiangiogenic therapy and may provide a potential therapeutic target for liver cancer treatment.

## 1. Introduction

Because vascular endothelial cells are genetically stable, it was once assumed that antiangiogenic therapy targeting endothelial cells would not become resistant. However, evidences from preclinical and clinical studies demonstrate that cancer cells can also acquire resistance to antiangiogenic agents. A variety of possible mechanisms, such as producing redundant angiogenic factors following anti-VEGF therapies, recruiting new vessels by vessel cooption or vasculogenic mimicry, and producing more invasive and metastatic tumor cells, have been elucidated [1–3]. Cancer stem cells (CSCs), also termed cancer initiating cells, are a small subgroup of cancer cells that have the ability to self-renew and differentiate to diverse cells that comprise the tumor. These cells are believed to be the primary cause of cancer recurrence and chemotherapy-resistance [4–6]. Recently, studies have shown that CSCs from some cancers, such as glioblastoma [7–10], breast cancer [11], and ovarian cancer [12], are able to transdifferentiate into endothelial cells and further form functional blood vessels. Because the process of this new vessel formation is VEGF-independent, it is resistant to the anti-VEGF therapy, indicating another possible resistance mechanism to antiangiogenic agents.

Liver cancer is the sixth most commonly diagnosed cancer and the fourth leading cause of cancer death worldwide. It is estimated that there are 841,000 new liver cancer cases and 782,000 deaths during 2018 [13]. Despite significant advances in combined therapies, hepatocellular carcinoma (HCC) is still characterized by a poor prognosis and a low survival rate during the past decades, due to recurrence and metastasis, as well as the failure of chemo- and radiotherapy [14]. Because HCC is a highly vascularized tumor, anti-angiogenesis therapies have become the standard care for recurrent and unresectable HCC. Unfortunately, the efficacy is modest and limited. The recurrence rate and mortality have not been significantly improved [15, 16]. Studies have showed that endothelial cells from HCC possess increased angiogenic activity and resistance to chemotherapeutic drugs and angiogenesis inhibitors [17]. However, it is not clear whether there exist a proportion of CSCs-differentiated endothelial cells in HCC, which results in the resistance to antiangiogenic therapy.

Reactive oxygen species (ROS), acting as signaling molecules, can regulate the proliferation, differentiation, and apoptosis of various cancer cells [18]. Studies also demonstrate that ROS play important roles in stem cells. For example, it has been shown that hematopoietic stem cells contain lower levels of ROS than their more mature progeny and that these differences appear to be critical for maintaining stem cell function [19]. Accordingly, increasing the ROS level in hematopoietic stem cells by knockout the gene of Atm or FoxO may promote the differentiation of hematopoietic stem cells [20, 21]. These results indicate that ROS play an important role in regulating the differentiation of hematopoietic stem cells. Similar to hematopoietic stem cells, CSCs also contain lower levels of ROS than non-CSCs, which may be due to reduced ROS production and/or enhanced ROS scavenging systems in the CSCs [22]. However, whether ROS regulate endothelial differentiation of CSCs is not known yet. So we ask whether there exists a new angiogenic pattern, in which the endothelial cells of HCC are derived from the transdifferentiation of CSCs and whether this process is regulated by ROS. To test the hypothesis, we examined the ability of liver cancer stem-like cells to transdifferentiate into endothelial cells and the regulating role of ROS on this process.

## 2. Materials and Methods

### 2.1. Chemicals and Reagents

Dulbecco's modified Eagle's medium (DMEM) and DMEM/F12 medium were obtained from Gibco (Thermo Fisher Scientific, Inc. Waltham, MA, USA). Fetal bovine serum (FBS) was purchased from National HyClone Bio-Engineering Co., Ltd. (Lanzhou, China). Recombinant human vascular endothelial growth factor (VEGF), basic fibroblast growth factor (bFGF), and epidermal growth factor (EGF) were purchased from PeproTech (Rocky Hill, NJ, USA). B27 supplement without vitamin A and N2 supplement were purchased from Invitrogen (Carlsbad, CA, USA). Growth factor-reduced Matrigel was purchased from Becton Dickinson (Bedford, MA, USA). MCDB131 medium, hydrocortisone, sulforhodamine B (SRB), 2′,7′-dichlorodihydrofluorescein diacetate (DCFH-DA), N-Acetyl-L-cysteine (NAC), *γ*-secretase inhibitor (DAPT), Akt1/2 kinase inhibitor, and L-Buthionine-sulfoximine (BSO) were purchased from Sigma (St. Louis, MO, USA). Bevacizumab was purchased from Roche Pharma (Schweiz) Ltd. (South San Francisco, CA, USA). I*κ*B phosphorylation inhibitor (BAY 11-7082) was obtained from Beyotime Biotechnology (Shanghai, China). Primary antibodies directed against I*κ*B kinase (IKK; ab178870) and p-IKK (ab55341), rabbit polyclonal to CD31 (ab32457), and mouse monoclonal (4F9) to Von Willebrand Factor (vWF, ab20435) were purchased from Abcam (Cambridge, MA, USA). Primary antibodies directed against protein kinase B (Akt; sc-8312), p-Akt (sc-7985-R), and *β*-actin (sc-130656) were purchased from Santa Cruz Biotechnology, Inc. (Dallas, TX, USA). The secondary antibodies of Alexa Fluor®594-conjugated AffiniPure Goat Anti-Rabbit IgG (H+L) and Alexa Fluor®488-conjugated AffiniPure Goat Anti-Mouse IgG (H+L) were purchased from Jackson ImmunoResearch Laboratories, Inc. (West Grove, PA, USA). Horseradish peroxidase-conjugated goat anti-rabbit IgG (ZB-2301) was purchased from ZSGB-BIO (Beijing, China).

### 2.2. Cell Culture

Human hepatocellular carcinoma cell line Hep G2 cells were cultured in DMEM supplemented with 10% FBS. Human microvascular endothelial cell line HMEC-1 cells were cultured in MCDB131 medium supplemented with 1.18 mg/ml NaHCO_3_, 20% inactivated FBS, 10 ng/ml EGF, and 1 *μ*g/ml hydrocortisone. All cells were cultured in a highly humidified atmosphere of 5% CO_2_ at 37°C.

### 2.3. Sphere Formation and Passage

Sphere was cultured as previously described with some modification [23]. That is, Hep G2 cells were collected, washed to remove serum, and then suspended in serum-free DMEM/F12 medium supplemented with 10 ng/ml bFGF, 20 ng/ml EGF, 2% B27, and 1% N2 supplement. Subsequently, the cells were cultured in ultralow attachment 6-well plates at a density of 2,000 cells/ml in 2 ml culture medium. Sphere formation was then tracked on days 1, 3, 5, 7, 9, and 11 under an inverted microscope. To propagate spheres, the spheres were collected, dissociated with trypsin, and resuspended in serum-free DMEM/F12 medium. The dissociated single cells were then similarly transferred into ultralow attachment 6-well plates as mentioned above. The third passage of sphere cells was collected for the following experiments.

### 2.4. Endothelial Differentiation Assay

Sphere cells dissociated with trypsin were cultured in MCDB131 complete endothelial medium as described in Section 2.2 with or without 10 ng/ml VEGF and passaged when they reached 95% confluence. After being passaged for two generations, cells were collected and regarded as endothelial differentiated sphere cells for the following experiments.

### 2.5. Immunofluorescence Assay

Cells were fixed with methyl and blocked with 1% BSA. The primary antibodies, including rabbit polyclonal to CD31 and mouse monoclonal to vWF, were added and incubated overnight at 4°C. After washing with PBS, the secondary antibodies labeled with fluorescent dyes were added and incubated at room temperature for 1 h. Then, the cells were counterstained with DAPI, and images were captured under a fluorescent microscope.

### 2.6. Tube Formation

Cells were seeded at a density of 7.5 × 10^4^ per well in 100 *μ*l MCDB 131 medium on 96-well plates precoated with Matrigel. After incubated for 24 hours, images were captured under an inverted microscope.

### 2.7. Cell Viability Assay

Cell viability was determined by SRB assay with some modifications [24]. Endothelial differentiated sphere cells were plated into 96-well plates at a density of 1 × 10^4^ per well in 100 *μ*l culture medium. After incubation overnight, cells were treated with different concentrations inhibitors for 24 h. The cultures were fixed with cold 10% trichloroacetic acid at 4°C for 1 h and then washed with water. After the plates were air-dried, the fixed cells were stained with 0.4% SRB for 10 min and washed repeatedly with 0.1% acetic acid to remove the unbound dye. The bound SRB was dissolved in 1% Tris base solution (pH 10.5). The optical density was measured at 510 nm on a microplate reader.

### 2.8. ROS Assay

To study intracellular ROS levels, cells were stained with 10 *μ*M DCFH-DA at 37°C for 30 minutes. Subsequently, cells were rinsed with D-Hanks' solution to remove the excess dye. Then, the cells were resuspended in D-Hanks' solution and immediately analyzed by flow cytometry.

### 2.9. Western Blotting

After treatments, cells were lysed with RIPA buffer supplemented with proteinase inhibitors. Equal amounts of protein from each sample were resolved by 6%-10% SDS polyacrylamide gels and then transferred onto the polyvinylidene fluoride (PVDF) membrane. After blocking with 5% fat-free milk, the membrane was incubated with the primary antibody at 4°C overnight, followed by exposure to a horseradish peroxidase-conjugated secondary antibody for 2 h. Then, the protein bands were visualized using enhanced chemiluminescence reagents on X-ray film.

### 2.10. Statistical Analyses

Results were expressed as mean ± SD. Statistical analyses were performed by Student's *t*-test and ANOVA using the SPSS version 20 statistical analysis package (SPSS Inc., Chicago, IL, USA). *P* value less than 0.05 was considered significant.

## 3. Results

### 3.1. Hep G2 Cells Could Form Spheres

**Figure 1:**
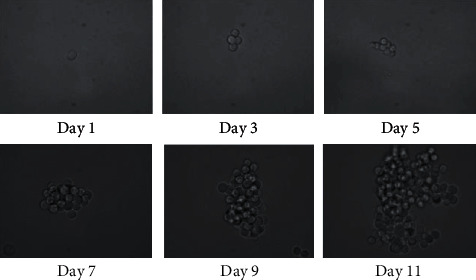
Hep G2 cells form nonadherent, self-renewing spheres in stem cell-conditioned medium. The sphere formation of a single cell was recorded at days 1, 3, 5, 7, 9, and 11 (200×).

After being plated in sphere induction medium in ultra-low attachment 6-well plates, Hep G2 cells grew in an anchorage-independent fashion and formed spheres 7-9 days later. When the spheres were passaged, cells dissociated from spheres could form new spheres again.  Figure 1 recorded the process of single Hep G2 cell forming a sphere.

### 3.2. Sphere Cells Differentiate to Endothelial Cells

**Figure 2:**
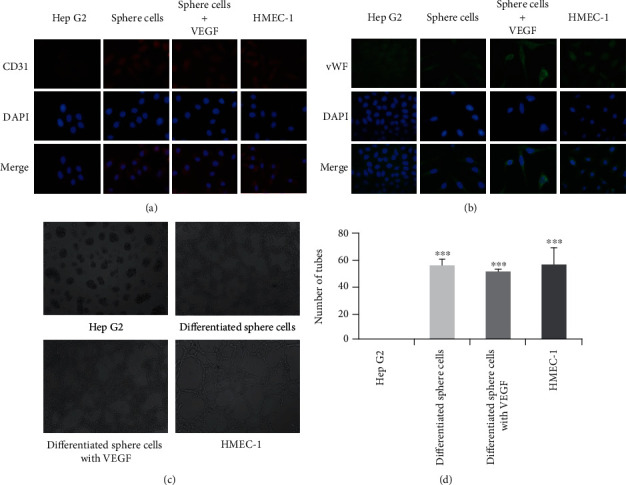
Cancer stem-like sphere cells differentiate to endothelial cells. Cells were cultured in MCDB131 endothelial medium with or without VEGF (10 ng/ml) for two generations. Expressions of endothelial markers CD31 (a) and vWF (b) were detected with immunofluorescence staining. The cell nuclei were counterstained with DAPI (200 ×). (c) Formation of capillary-like structure on Matrigel (100×). (d) A number of formed tubes were calculated. The data were presented as mean ± SD (*n* = 3). ^∗∗∗^*P* < 0.001 vs. Hep G2 group.

As shown in Figure 2, after cultured in MCDB131 endothelial medium for two generations, adherent Hep G2 cells did not express endothelial markers, including CD31 and vWF. In contrast, following the same culture conditions as Hep G2 cells, sphere cells, now regarded as endothelial differentiated sphere cells, expressed both endothelial markers. Moreover, addition of 10 ng/ml VEGF in MCDB131 endothelial medium did not affect the CD31 and vWF expression of sphere cells. Similar to sphere cells, HMEC-1 cells, which were selected as a positive control, also expressed both CD31 and vWF under the same culture conditions. Formation of capillary-like structures on Matrigel is one of the widely used functional tests for endothelial cells. We then examined the ability of tube formation with tube formation assay. After seeded on Matrigel for 24 h, adherent Hep G2 cells failed to form tube structures. However, both endothelial differentiated sphere cells and HMEC-1 cells developed tube structures. Addition of VEGF (10 ng/ml) did not affect the tube formation of endothelial differentiated sphere cells. All these results indicated that sphere cells from Hep G2 have the ability to differentiate into endothelial cells.

### 3.3. The Endothelial Differentiation of Sphere Cells Is Independent of VEGF and NOTCH Signaling but Dependent on the Activation of Akt and IKK

**Figure 3:**
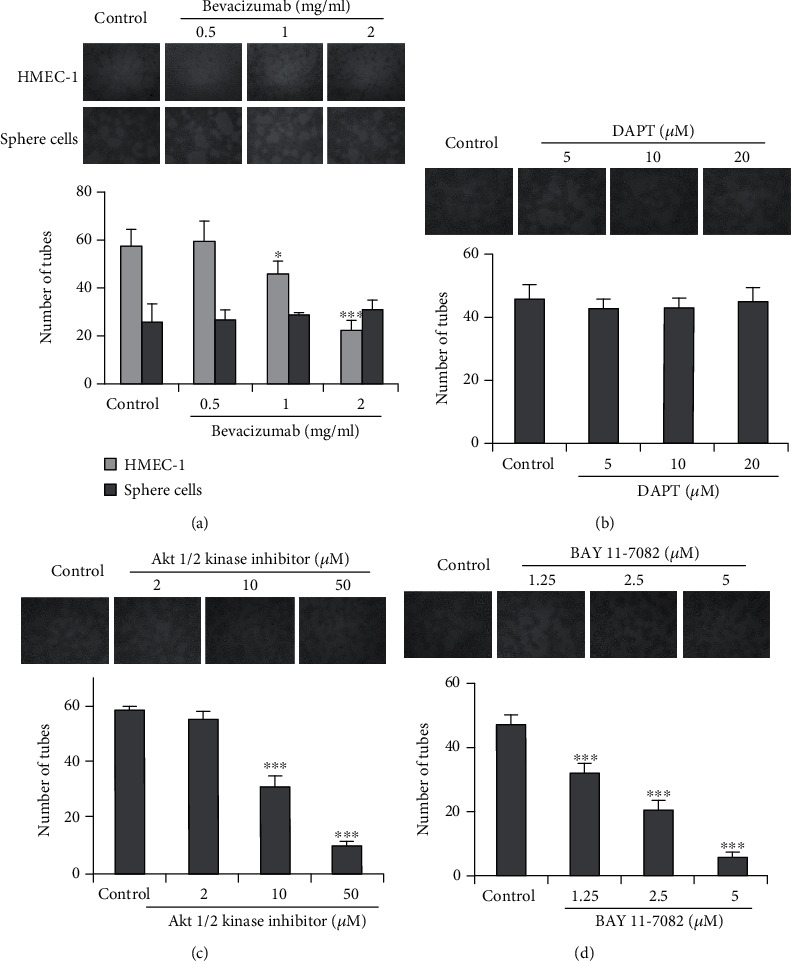
Effects of various signaling pathway inhibitors on the tube formation of HMEC-1 or endothelial differentiated sphere cells on Matrigel. HMEC-1 or endothelial differentiated sphere cells seeded on Matrigel were treated with or without indicated concentrations of Bevacizumab (a), DAPT (b), Akt1/2 kinase inhibitor (c), or BAY 11-7082 (d) for 24 h. The photographs of tube formation were taken (100×). The number of formed tubes was calculated. The data were presented as mean ± SD (*n* = 3). ^∗^*P* < 0.05, ^∗∗∗^*P* < 0.001 vs. control group.

**Figure 4:**
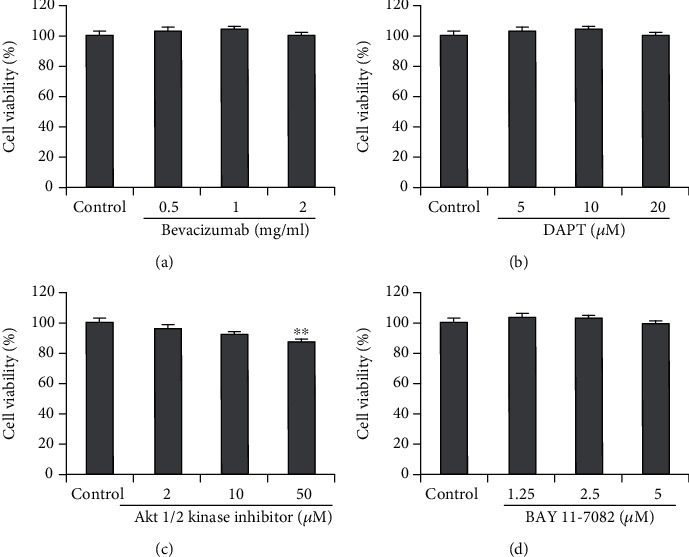
Effects of various signaling pathway inhibitors on the cell viability of endothelial differentiated sphere cells. Endothelial differentiated sphere cells were treated with or without indicated concentrations of Bevacizumab (a), DAPT (b), Akt1/2 kinase inhibitor (c), or BAY 11-7082 (d) for 24 h. Cell viability was measured with SRB assay. The data were presented as mean ± SD (*n* = 6). ^∗∗^*P* < 0.001 vs. control group.

To further explore the underling mechanisms of endothelial differentiation of sphere cells, we investigated the impacts of Bevacizumab, a VEGFA-binding antibody currently in clinical use, DAPT, a *γ*-secretase inhibitor that effectively inhibits Notch signaling, Akt1/2 kinase inhibitor, and BAY 11-7082, an IKK-*β* inhibitor, on the tube formation of endothelial differentiated sphere cells. As shown in Figure 3, exposure to 1, 2 mg/ml Bevacizumab did not affect the tube formation of endothelial differentiated sphere cells, yet it blocked the tube formation of HMEC-1 cells. These results, combined with the above results that addition of VEGF did not affect the CD31 and vWF expression and tube formation of endothelial differentiated sphere cells, indicated that sphere cells differentiate into endothelial cells in a VEGF-independent manner. Likewise, treatment with DAPT at the concentrations of 5, 10, and 20 *μ*M did not affect the tube formation of endothelial differentiated sphere cells. In contrast, addition of Akt1/2 kinase inhibitor (2, 10, 50 *μ*M) resulted in significant suppression of tube formation of endothelial differentiated sphere cells. Moreover, the same inhibition results were also observed when we treated endothelial differentiated sphere cells with different concentrations of BAY 11-7082. In addition, to determine whether the effects of Bevacizumab, DAPT, Akt1/2 kinase inhibitor, and BAY 11-7082 on the tube formation were related to their cytotoxicity, we then examined their effects on cell viability of endothelial differentiated sphere cells. As shown in Figure 4, treatment with Bevacizumab, DAPT, Akt1/2 kinase inhibitor at 2 and 10 *μ*M, and BAY 11-7082 did not affect the viability of endothelial differentiated sphere cells, indicating that their effects on tube formation were not due to the cytotoxicity. Akt1/2 kinase inhibitor at 50 *μ*M inhibited the viability of endothelial differentiated sphere cells with an inhibition rate of 12.8%. Since this inhibitory effect on cell viability was much weaker than that of the tube formation, indicating that the effect of 50 *μ*M Akt1/2 kinase inhibitor on the tube formation was also not the result of its cytotoxic effect. These results indicated that sphere cells are able to differentiate into endothelial cells in a VEGF- and NOTCH-independent, but an Akt- and IKK-dependent manner.

### 3.4. Hydrogen Peroxide Promotes the Differentiation of Sphere Cells into Endothelial Cells

**Figure 5:**
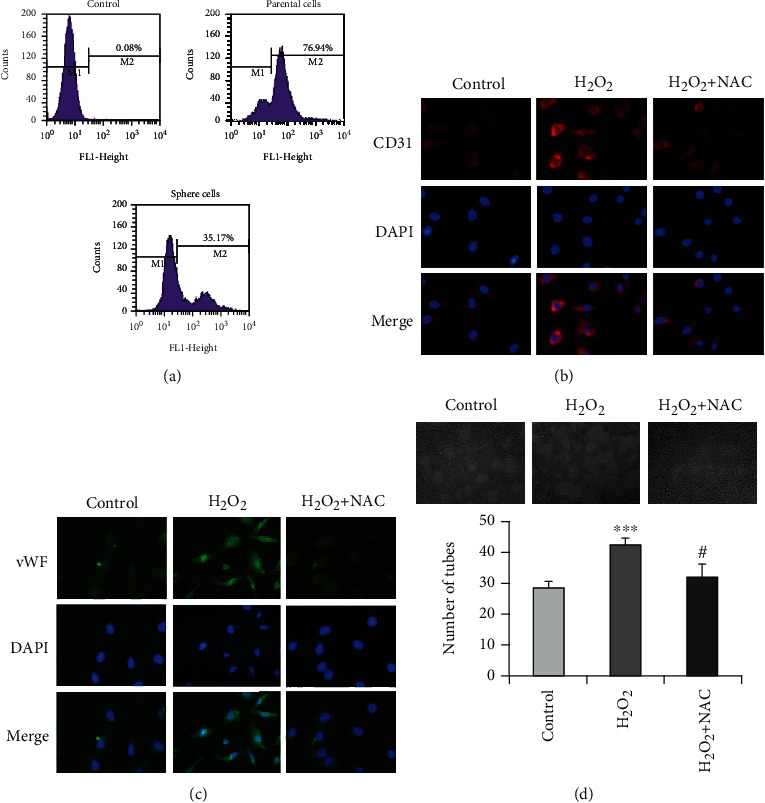
Hydrogen peroxide promotes the endothelial differentiation of sphere cells. (a) ROS levels in parental Hep G2 and sphere cells were examined with DCFH-DA staining and analyzed by flow cytometry. Endothelial differentiated sphere cells cultured in the presence or absence 1 mM NAC for 30 min were further treated with 100 *μ*M H_2_O_2_ for 24 h. The cells were then subjected to immunofluorescence analysis for the expression of CD31 (b) and vWF (c) (200×). (d) Endothelial differentiated sphere cells were seeded on Matrigel in the presence or absence of NAC (1 mM) for 30 min and further treated with or without 100 *μ*M H_2_O_2_ for 24 h. The photographs of tube formation were taken (100×). The number of formed tubes was calculated. The data were presented as mean ± SD (*n* = 3). ^∗∗∗^*P* < 0.001 vs. control group and #*P* < 0.05 vs. H_2_O_2_ treatment alone.

It has been reported that liver cancer stem cells normally contain low levels intracellular ROS, and ROS also play an important role in the processes of self-renewal and differentiation of cancer stem cells [25, 26]. So we first measured the intracellular ROS levels in sphere cells and their parental Hep G2 cells using DCFH-DA staining. As shown in Figure 5(a), sphere cells contained lower levels of intracellular ROS than their parental cells. Next, to determine whether ROS are involved in the process of endothelial differentiation of sphere cells, we treated the endothelial differentiated sphere cells with 100 *μ*M hydrogen peroxide in the presence or absence of NAC (1 mM), which acts as a ROS scavenger by promoting intracellular biosynthesis of glutathione (GSH). As shown in Figures 5(b)–5(d), treatment with hydrogen peroxide for 24 h increased the CD31 and vWF expression of endothelial differentiated sphere cells. Under this treatment condition, hydrogen peroxide also increased the tube formation of endothelial differentiated sphere cells. Importantly, pretreatment with NAC almost totally canceled the hydrogen peroxide-induce increase of the CD31 and vWF expression and tube formation. These results suggest that hydrogen peroxide could promote the endothelial differentiation of sphere cells.

### 3.5. L-Buthionine Sulfoximine (BSO) Promotes the Differentiation of Sphere Cells into Endothelial Cells

**Figure 6:**
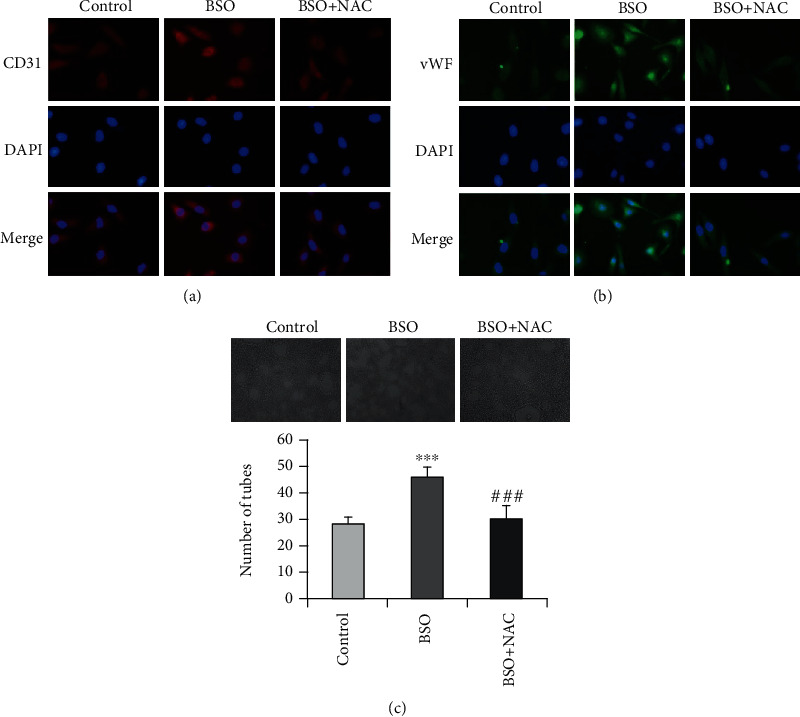
BSO promotes the endothelial differentiation of sphere cells. Endothelial differentiated sphere cells cultured in the presence or absence of 1 mM NAC for 30 min were further treated with 1 mM BSO for 24 h. The cells were then subjected to immunofluorescence analysis for the expression of CD31 (a) and vWF (b) (200×). (c) Endothelial differentiated sphere cells were seeded on Matrigel in the presence or absence of 1 mM NAC for 30 min and then further treated with or without 1 mM BSO for 24 h. The photographs of tube formation were taken (100×). The number of formed tubes was calculated. The data were presented as mean ± SD (*n* = 3). ^∗∗∗^*P* < 0.001 vs. control group and ^###^*P* < 0.001 vs. BSO treatment alone.

Studies have shown that lower ROS levels in cancer stem cells are associated with increased expression of free radical scavenging systems. Therefore, treatment of cancer stem cells with BSO, an inhibitor of GSH biosynthesis, results in the depletion of GSH levels, and thus the accumulation of intracellular ROS [22]. To further explore the effect of endogenous ROS on the endothelial differentiation of sphere cells, we treated endothelial differentiated sphere cells with 1 mM BSO in the presence or absence of NAC (1 mM). As shown in Figure 6, exposure of endothelial differentiated sphere cells to BSO increased the CD31 and vWF expression. BSO treatment also induced the tube formation of endothelial differentiated sphere cells. Importantly, all these changes caused by BSO were abolished when the intracellular ROS were scavenged by NAC. Thus, the results suggest that elevating the intracellular ROS level by inactivating the antioxidant defense also induces the differentiation of sphere cells into endothelial cells.

### 3.6. Akt and IKK Activation Is Required for ROS-Mediated Endothelial Differentiation of Sphere Cells

**Figure 7:**
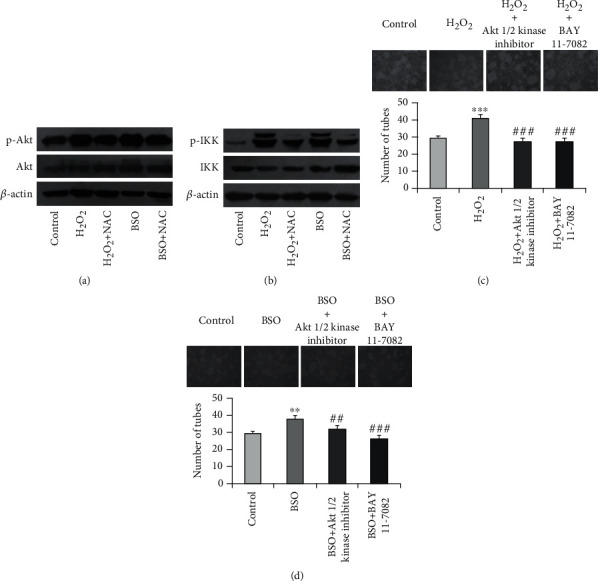
ROS induced endothelial differentiation of sphere cells through the activation of Akt/IKK signaling pathway. Endothelial differentiated sphere cells cultured in the presence or absence 1 mM NAC for 30 min were further treated with 100 *μ*M H_2_O_2_ or 1 mM BSO for 24 h. The cells were then subjected to western blotting for detecting the indicated proteins (a, b). Endothelial differentiated sphere cells were seeded on Matrigel in the presence or absence of Akt1/2 kinase inhibitor (10 *μ*M) or BAY 11-7082 (2.5 *μ*M) for 30 min and then further treated with or without 100 *μ*M H_2_O_2_ (c) or 1 mM BSO (d) for 24 h. The photographs of tube formation were taken (100×). The number of formed tubes was calculated. The data were presented as mean ± SD (*n* = 3). ^∗∗^*P* < 0.01, ^∗∗∗^*P* < 0.001 vs. control group and ^##^*P* < 0.01, ^###^*P* < 0.001 vs. H_2_O_2_ or BSO treatment alone.

As described above, the endothelial differentiation of sphere cells was Akt- and IKK-dependent. To clarify whether the promoting action of hydrogen peroxide and BSO on endothelial differentiation was related to the activation of both Akt and IKK, we first examined the effects of hydrogen peroxide or BSO on the activation of both Akt and IKK with western blotting. As shown in Figure 7(a), treatment with 100 *μ*M hydrogen peroxide or 1 mM BSO did not affect the expression of total Akt but significantly increased the phosphorylation of Akt in endothelial differentiated sphere cells. The increase of Akt phosphorylation induced by hydrogen peroxide or BSO could be abolished by the pretreatment with 1 mM NAC. Likewise, treatment with 100 *μ*M hydrogen peroxide or 1 mM BSO did not affect the level of total IKK but increased the level of p-IKK. This increase of p-IKK was reversed by NAC (1 mM) (Figure 7(b)). Because IKK activation is one of the downstream events of Akt activation, these results indicated that ROS treatment activated the Akt/IKK signaling pathway. Next, to determine whether Akt/IKK signaling is indeed involved in ROS-induced endothelial differentiation of sphere cells, we explored the effect of Akt1/2 kinase inhibitor and BAY 11-7082 on the ROS-induced endothelial differentiation of sphere cells with tube formation assay. As shown in Figures 7(c) and 7(d), pretreatment with Akt1/2 kinase inhibitor (10 *μ*M) or BAY 11-7082 (2.5 *μ*M) inhibited hydrogen peroxide or BSO-induced tube formation of endothelial differentiated of sphere cells. These results suggest that Akt/IKK signaling pathway is involved in the ROS-induced differentiation of sphere cells into endothelial cells.

## 4. Discussion

Cancer stem cells are a small portion of cancer cells with the feature of stem cells. They have self-renewal and differential capacity and have been considered as the origin of tumor recurrence and metastasis. They are also responsible for the resistance to chemotherapy and radiotherapy in clinic. Currently, commonly used cell surface markers for isolating liver CSCs include CD133, CD90, CD44, epithelial cell-adhesion molecule (EpCAM), CD13, OV6, and ALDH [27]. However, none of these markers are specific for liver CSC. There is no generally accepted surface marker for liver CSC [28]. Sphere formation assay is a functional approach commonly used to identify CSCs and study their properties [29]. Studies have shown that sphere cells enriched from HCC cell lines and primary tumor cells exhibited CSC properties, including proliferation, self-renewal, drug resistance, lower ROS levels, and high tumorigenicity [23, 26, 30]. More importantly, as Ma et al. reported, the cell surface marker selection only enriches one CSC subpopulation, while sphere-forming culture enriches different subpopulations of CSCs with certain HCC biomarkers, and thus the most complete CSC population from a bulk tumor [30]. Therefore, we finally choose sphere formation assay to isolate cancer stem-like cells in our study.

Recently, it is reported that glioblastoma [7–10], breast [11], and ovarian [12] CSCs have the ability to differentiate into endothelial cells both morphologically and functionally. Studies from Marfels et al. also demonstrated that chemoresistant hepatocellular carcinoma cells showed increased pluripotent capacities and the ability to transdifferentiate into functional endothelial-like cells both *in vitro* and *in vivo* [31]. However, another report from Ghanekar et al. showed that endothelial cells did not arise from tumor-initiating cells in human hepatocellular carcinoma [32]. But Yao et al. pointed out that the conclusion made by Ghanekar et al. was not convincing due to too little data and the uncommon method they used to identify CSCs [33]. These mean whether liver CSCs have the ability to differentiate into endothelial cells remains unclear. In the present study, we examined the ability of liver CSCs to transdifferentiate into endothelial cells and explored the role of ROS in regulating this process, together with the molecular mechanism involved therein. Our results suggested that cancer stem-like sphere cells enriched from Hep G2 are able to differentiate into endothelial cells.

It is well documented that the differentiation process of CSCs involves multiple signal pathways [34]. Different type of CSCs may activate distinct signal pathways during the endothelial differentiation process. For example, stem-like ovarian cancer cells differentiate into endothelial cells in a VEGF-independent but IKK*β*-dependent manner [12]. Similarly, endothelial differentiation of other CSCs such as glioblastoma [9], breast cancer [11], and renal carcinoma [35] is all VEGF-independent. Moreover, endothelial differentiation of glioblastoma CSCs is also dependent on NOTCH signaling pathway [8]. In our study, neither Bevacizumab nor DAPT inhibits the tube formation of endothelial differentiated sphere cells, indicating that endothelial differentiation of cancer stem-like sphere cells from Hep G2 was independent of VEGF and NOTCH signaling.

Akt is a serine/threonine protein kinase. Once activated, Akt regulates many physiological cell processes including proliferation, differentiation, apoptosis, and motility through phosphorylating a series of protein substrates [36]. Recent studies have revealed that PI3K/AKT signaling pathway is upregulated in CSCs, which is related to the maintenance of CSCs phenotype in several tumors [37]. NF-*κ*B is a transcription factor. In resting cells, NF-*κ*B is sequestered in cytosol via interaction with I*κ*B. Once activated by upstream signaling molecules, I*κ*B kinase (IKK) phosphorylates I*κ*B, tagging it for degradation. As a result, NF-*κ*B is liberated from I*κ*B, translocates to the nucleus, and proceeds to activate the expression of a cohort of target genes which are related to a series of cellular processes, including the inflammatory response, cellular adhesion, differentiation, proliferation, autophagy, senescence, and apoptosis [38]. Elevated or constitutive NF-*κ*B activity, which has been found in CSCs from many types of cancers, participates in the self-renewal, proliferation, survival, and differentiation of CSCs [39]. In liver CSCs, NF-*κ*B signaling is frequently activated [40]. Based on the fact, we then explored the effect of Akt1/2 kinase inhibitor and BAY 11-7082, an IKK-*β* inhibitor, on the tube formation of endothelial differentiated sphere cells. We found that either Akt1/2 kinase inhibitor or BAY 11-7082 dose-dependently blocked tube formation of endothelial differentiated sphere cells, suggesting that sphere cells from Hep G2 differentiate into endothelial cells in an Akt- and IKK-dependent manner. Collectively, our findings provided an important evidence that liver CSCs have the ability to differentiate into endothelial cells, as well as the underlying mechanisms of this process.

Studies have shown that CSCs contain lower levels ROS, which contributes to their stemness and resistance to chemotherapy and radiation therapy [41]. In our experiments, cancer stem-like sphere cells derived from Hep G2 also have lower levels of ROS than nonsphere cells, which are consist with the previous reports from Haraguchi et al. [25].

Accumulative evidences have demonstrated that ROS and ROS-dependent signaling pathways play an important role in self-renewal and differentiation of CSC. Sato et al. reported that ROS promote the differentiation of glioma-initiating cells through the activation of p38 MAPK signaling pathway [42]. However, it remains poorly explored whether ROS are involved the transdifferentiation process of cancer stem cells into endothelial cells. In the present study, we found that both exposure to exogenous ROS and elevating endogenous ROS by inhibiting the cellular antioxidant mechanism promote endothelial differentiation of cancer stem-like sphere cells. These indicate that ROS play an essential role in regulating the differentiation of liver cancer stem cells into endothelial cells.

Emerging evidences indicate that ROS play an essential role in the process of self-renewal and differentiation of CSCs through the activation of multiple ROS-dependent signaling pathways, such as PI3K/AKT, ATM, and Notch pathway [43]. Our results indicate that Akt/IKK signaling pathway is critical for the differentiation cancer stem-like sphere cells into endothelial cells. So we then examined the effects of ROS on the activation Akt/IKK signaling pathway. We found that treatment with either hydrogen peroxide or BSO induced the phosphorylation of both Akt and IKK. This increased phosphorylation could be blocked by NAC. Accordingly, both Akt1/2 kinase inhibitor and BAY 11-7082 blocked the tube formation induced by hydrogen peroxide or BSO. These data suggest that ROS promote the differentiation of cancer stem-like sphere cells into endothelial cells through the activation of Akt/IKK signaling pathway.

However, it is important to note that although many studies have confirmed the CSC properties of sphere-formed cells from liver cancer [23, 26, 30], it would be more convincing to examine some of CSC markers such as CD133 and CD44 variant expressions and tumorigenicity of sphere-formed cells to validate the CSC phenotype. In addition, sphere cells in the present study were isolated only in one HCC cell line Hep G2, which is thought to be derived from hepatoblastoma. Other HCC-derived cell lines will be needed in the future to further confirm the findings.

## 5. Conclusions

Collectively, the results of the present study demonstrate that cancer stem-like sphere cells from Hep G2 are able to differentiate into endothelial cells both morphologically and functionally, and this process is independent of VEGF and NOTCH signaling but dependent on the activation of Akt and IKK. ROS promote endothelial differentiation of cancer stem-like sphere cells through activation of Akt/IKK signaling pathway. Therefore, our study reveals a new mechanism of angiogenesis in liver cancer that might be related to the resistance to conventional antiangiogenic treatments. Moreover, our data also indicate that targeting ROS-dependent Akt/IKK signaling pathway may provide a novel therapeutic strategy for liver cancer treatment.

## Data Availability

The data used to support the findings of this study are included within the article.
